# Dynamic distribution of conventional dendritic cells in the lung, blood and spleen from the early phase of sepsis-induced acute lung injury

**DOI:** 10.1186/cc9624

**Published:** 2011-03-11

**Authors:** J Liu, H Qiu

**Affiliations:** 1 Zhong-Da Hospital, Southeast University, Nanjing, China

## Introduction

Respiratory dendritic cells (DCs), especially conventional DCs, are centrally involved in the induction phase of the immune response in our respiratory system. However, their role in acute lung injury (ALI) is largely unknown and little information concerning cDCs of blood and spleen is available on ALI.

## Methods

c57BL/6 mice were intratracheally challenged with *Escherichia coli *LPS (2 mg/kg). At 6 hours, 12 hours, and 24 hours after i.t. delivery of LPS (ALI group) or PBS alone (Control group), mice were sacrificed, and blood, lungs and spleens were collected. cDCs were detected using flow cytometry in enzyme-digested lung, blood, and spleen.

## Results

The sepsis-induced ALI showed divergent kinetics of cDCs in peripheral blood, lung and spleen, respectively. ALI resulted in a rapid cDC accumulation in the lung, the frequencies of cDCs in ALI mice were significantly increased during all time points, compromised (2.38 ± 0.78)% at 12 hours, and peaked at 24 hours postchallenge (2.86 ± 0.55)%, relative to lung nucleated cells (*P *< 0.05 vs. Control). However, splenic cDCs only showed a markedly transient augmentation to a peak (1.92 ± 0.25)% at 12 hours (*P *< 0.05 vs. Control), but subsequently declined to baseline (0.96 ± 0.21)% at 24 hours. In contrast to the lung cDC accumulation at 6 hours, sepsis-induced ALI led to a decreased percentage (0.32 ± 0.10)% of circulating cDCs at the same time point (*P *< 0.05 vs. Control), then the percentage of circulating cDCs was significantly increased (1.50 ± 0.31)% compared with that of control mice at 12 hours, and further increased (2.20 ± 0.92)% at 24 hours after LPS-induced ALI (*P *< 0.05 vs. Control). All cDCs within the blood, lungs and spleens had undergone a modest maturation in ALI from sepsis.

## Conclusions

ALI by sepsis produces different quantitative and phenotypical changes in pulmonary, circulatory and splenic cDCs. Lung cDCs may participate in the early inflammatory response to ALI.

